# Recycled Materials in Civil Engineering Application

**DOI:** 10.3390/ma16227075

**Published:** 2023-11-08

**Authors:** Malgorzata Ulewicz

**Affiliations:** Faculty of Civil Engineering, Czestochowa University of Technology, Dabrowskiego 69 Street, 42-201 Czestochowa, Poland; malgorzata.ulewicz@pcz.pl; Tel.:+48-343250935

In recent years, the construction sector has shown great interest in the use of various by-products and industrial waste, as well as the consumer products used. The use of various waste and recycled materials by the construction sector fits well into the idea of sustainable construction. Replacing at least partial natural resources with waste materials allows them to be preserved for future generations and is also the first step toward a circular economy. It should be remembered that the design and production of building materials should take into account appropriate standards and environmental protection requirements. Moreover, the use of waste from various industries and recycling materials in building materials must be preceded by a series of laboratory tests to make them harmless to the environment and people.

This Special Issue of “Recycled Materials in Civil Engineering Application” publishes the latest results of scientific research on the use of waste and recycled material for the production of mortar, concrete, ceramic and other building material. Research in this area was conducted in many centers and universities around the world, including the USA, France, Pakistan, Turkey, Poland, Ukraine, Croatia, Brazil, China, Armenia and Chile. In this issue, twelve original scientific papers and two review articles were published. The topics of these articles cover a number of aspects that are important for construction and the environment, including determining the physicochemical and mechanical properties of mortar, concrete and ceramic materials produced from waste and recycled materials. A model for predicting the hydration process of a new alternative binder is also proposed, and methods and technologies for removing excess fluorine from various wastes, which are beneficial to the environment, are presented. Both laboratory research results and interesting and practical solutions are presented.

Zhao et al. [1] present the influence of adding glass fiber-reinforced plastic (GFRP) waste (in the range of 1–5%) on the properties of concrete. It has been shown that the addition of GFRP powder does not have a significant effect on the mechanical properties of concrete, while the addition of a small amount of GFRP cluster slightly improves the compressive and tensile strength of concrete. Pietrzak and Ulewicz [2] determined the influence of this addition (in the range of 2.5–10%) of post-consumer thermoplastic elastomer (TPE) derived from used car mats on the physical and mechanical properties of concrete. It has been shown that only the addition of post-consumer elastomer waste in an amount of up to 2.5% of the cement mass can be used as a replacement for sand and gravel aggregate in concrete without reducing its mechanical strength and microstructure. In turn, Kalak et al. [3] determined the mechanical properties of concrete produced with the addition of fly ash from the thermal conversion of municipal sewage sludge (SSFA). A correlation was demonstrated between the mechanical strength of waste-modified concrete and various parameters, including the composition of concrete mixtures (amount of sand and gravel, cement, SSFA), water-to-cement ratio (w/c) and sand point. In the scope examined by the authors, the addition of SSFA did not reduce the compressive strength of the concrete produced, and despite the lack of legal regulations regarding the physicochemical properties of SSFA, this material could be used for concrete.

In the production of concrete, fly ash, slag and biosilica were also used. Jura and Ulewicz [4] determined the properties of concrete produced with the addition of fly ash from the combustion of wood-sunflower biomass in a fluidized bed boiler. It was shown that fly ash used in an amount of 10–30% can be added as a substitute for sand in the production of concrete without impairing its mechanical properties (compressive strength and resistance to low temperatures) compared to the control concrete as the heavy metal ions present in the ashes do not have a negative impact on the environment. By contrast, Blikharskyy et al. [5] determine the mechanical properties of concrete produced with the addition of ground-granulated blast furnace slag (GGBFS) and fly ash (FA) and the corrosion susceptibility of reinforcing bars. It was found that the degree of fragmentation of GGBFS and FA and their simultaneous combination impacted the kinetics of increasing the strength of concrete. The highest compressive strength (6.5% higher) was obtained from concrete containing 10% GGBFS and 10% FA. In these types of concrete, the protection of the steel reinforcement was not compromised due to the low degree of substitution of Portland cement and its tighter microstructure. In turn, Muradyan et al. [6] determined the effect of adding biosilica and two different methods of mixing it with Portland cement (mixing the dry ingredients directly and mixing after dissolving biosilica in water) on the compressive strength of cement mortars. It has been shown that both mixing methods give positive results, and the highest compressive strength is achieved by mortars with the addition of 10% biosilica.

Waste materials were also used to produce other building materials, including autoclaved bricks and ballast mixtures. Bork et al. [7] used cement bypass dust (CBPD) to produce autoclaved bricks. It has been shown that the amount of CBPD dust used in autoclaved products depends on the chemical composition of the dust and, in particular, on the content of free CaO. The modification of the traditional silica–calcium mixture with bypass dust changes its phase composition and introduces new phases into the system in the form of portlandite and sylvine. The use of CBPD as a lime substitute does not inhibit the formation of hydrated lime silicates, characteristic of autoclaved products. On the contrary, an increase in the share of waste dust in products results in the formation of denser structures with a greater degree of crystallization, i.e., tobermorite. The complete replacement of quicklime with CBPD contributes to increasing the compressive strength of the manufactured bricks (by 5%) while reducing their bulk density (by 5.3%). On the other hand, Kostrzewa-Demczuk et al. [8] present the influence of adding basalt powder waste on the physical and mechanical properties of manufactured lime–sand products (silicates). It has been shown that the use of basalt flour at an amount of 10% as a component of lime–sand product brings positive effects (a double increase in compressive strength compared to traditional silicate). Unfortunately, with the increase in the addition of waste basalt flour, a decrease in the performance parameters of the manufactured products was recorded, although these products still met the conditions for traditional silicate bricks. Sufian et al. [9] determined the influence of adding marble flour (in the range of 5–30%) as a clay substitute on the properties of the produced bricks. It was shown that, with the addition of marble flour, the compressive strength and bulk density of the bricks decreased, but their water absorption capacity and porosity improved. Bricks made with the addition of 5–20% marble flour have adequate compressive strength in relation to the values required by standards, and the addition of marble waste does not significantly affect the amount of salt effloresce occurring in the bricks. In turn, Dimter et al. [10] determined the mechanical properties (California Bearing Ratio, compressive and intermediate tensile strength and resistance to freeze/thaw cycles) of wood fly ash (WFA) sand mixtures produced with different amounts of cement. It has been shown that WFA has a significant stabilizing effect on the sand mixture and improves its load-bearing capacity. Adding a small amount of cement causes the hydraulic reaction in the stabilized mixture to be more intense, resulting in greater strength and better resistance to freezing. The test results have shown that by replacing part of the sand with the addition of WFA (30%), a mixture with greater strength (by 90.7%) was obtained compared to the mixture of sand and cement alone, which contributes to savings during the construction of the pavement structure.

This Special Issue also presents an innovative solution that is useful for cement producers. Boukhelf et al. [11] present an artificial neural network (ANN) model to predict the hydration process of a new alternative binder. This model overcomes the lack of input parameters of physical models, providing a realistic explanation with fewer inputs and fast calculations. The verification of this model was carried out for mortar produced on the basis of CEM I and CEM III cement with added glass powder as the cement substitution. The proposed model could be useful for cement producers as it facilitates the quick identification of different hydration modes of new binders, using only the heat of the hydration test as an input parameter. An innovative approach to demolition waste was presented by Xiao et al. [12]. The authors proposed a process for preparing foamed lightweight soil using alkaline-activated concrete flour derived from prefabricated waste. It is best to use concrete flour powder, fly ash and slag at the amount of 60, 20 and 20%, respectively, to prepare light soil. The lightweight soil proposed by the authors is convenient to build without compaction, and its production costs are lower compared to filling soil or reinforced soil.

This Special Issue also includes two interesting review articles. Dobiszewska et al. [13] present a comprehensive review of literature reports on the use of rock dust from various geological origins for the production of mortars and concrete. The influence of rock dust as a substitute for fine aggregate on the properties of cement composites, such as workability, segregation and bleeding, mechanical properties and the durability of hardened concrete and mortar, was analyzed and assessed. The use of environmentally friendly rock dust in a specified amount to replace fine aggregate in cement-based composites has been shown to improve many properties in both the fresh and hardened state. The authors draw attention to the lack of information on the influence of particle size distribution in stone dust itself and on the properties of concrete and mortar. A detailed analysis of the particle size distribution can help in the decision to use stone flour because it affects the internal structure and many related mechanical properties of the manufactured materials. On the other hand, reports by Olejarczyk et al. [14] show that the production of building materials from various wastes containing hazardous substances, including fluorine, is still very limited. However, this type of waste can be used as additives or admixtures for the production of new construction materials after undergoing “solidification/stabilization” processes. The authors proposed several methods for reducing the concentration of fluoride ions in waste and several materials that can be used as fluorine adsorbents while also taking into account the low costs of processing these wastes.

All articles published in this Special Issue have been peer-reviewed by renowned experts. As a guest editor, I would like to thank all authors for their valuable contribution to the development of building materials engineering and the reviewers for their comments and suggestions that significantly improved the quality of the published articles. I would also like to express my sincere thanks to the Section Managing Editor, Ms. Freda Zhang, for her kind assistance in preparing this Special Issue of the Journal.
